# Intrinsically‐Stretchable and Patternable Quantum Dot Color Conversion Layers for Stretchable Displays in Robotic Skin and Wearable Electronics

**DOI:** 10.1002/adma.202420633

**Published:** 2025-05-06

**Authors:** Kiwook Kim, Dong Ryong Kim, Dohyeon Kim, Hyeon Hwa Song, Seungmin Lee, Yonghoon Choi, Kyunghoon Lee, Gwang Heon Lee, Jinhee Lee, Hye Hyun Kim, Eonhyoung Ahn, Jae Hong Jang, Yaewon Kim, Hyo Cheol Lee, Yunho Kim, Soo Ik Park, Jisu Yoo, Youngsik Lee, Jongnam Park, Dae‐Hyeong Kim, Moon Kee Choi, Jiwoong Yang

**Affiliations:** ^1^ Department of Energy Science and Engineering Daegu Gyeongbuk Institute of Science and Technology (DGIST) Daegu 42988 Republic of Korea; ^2^ Graduate School of Semiconductor Materials and Devices Engineering Center for Future Semiconductor Technology (FUST) Ulsan National Institute of Science and Technology (UNIST) Ulsan 44919 Republic of Korea; ^3^ School of Chemical and Biological Engineering Institute of Chemical Processes Seoul National University Seoul 08826 Republic of Korea; ^4^ Center for Nanoparticle Research Institute for Basic Science (IBS) Seoul 08826 Republic of Korea; ^5^ Department of Materials Science and Engineering Ulsan National Institute of Science and Technology (UNIST) Ulsan 44919 Republic of Korea; ^6^ School of Energy and Chemical Engineering Ulsan National Institute of Science and Technology (UNIST) Ulsan 44919 Republic of Korea; ^7^ Department of Chemistry Hong Kong University of Science and Technology (HKUST) Kowloon SAR 999077 Hong Kong; ^8^ Department of Biomedical Engineering Ulsan National Institute of Science and Technology (UNIST) Ulsan 44919 Republic of Korea; ^9^ Energy Science and Engineering Research Center Daegu Gyeongbuk Institute of Science and Technology (DGIST) Daegu 42988 Republic of Korea

**Keywords:** quantum dot, robotic skin, stretchable color conversion layer, stretchable display, wearable electronics

## Abstract

Stretchable displays are essential components as signal outputs in next‐generation stretchable electronics, particularly for robotic skin and wearable device technologies. Intrinsically‐stretchable and patternable color conversion layers (CCLs) offer practical solutions for developing full‐color stretchable micro‐light‐emitting diode (LED) displays. However, significant challenges remain in creating stretchable and patternable CCLs without backlight leakage under mechanical deformation. Here, a novel material strategy for stretchable and patternable heavy‐metal‐free quantum dot (QD) CCLs, potentially useful for robotic skin and wearable electronics is presented. Through a versatile crosslinking technique, uniform and high‐concentration QD loading in the elastomeric polydimethylsiloxane matrix without loss of optical properties is achieved. These CCLs demonstrate excellent color conversion capabilities with minimal backlight leakage, even under 50% tensile strain. Additionally, fine‐pixel patterning process with resolutions up to 300 pixels per inch is compatible with the QD CCLs, suitable for high‐resolution stretchable display applications. The integration of these CCLs with micro‐LED displays is also demonstrated, showcasing their use in haptic‐responsive robotic skin and wearable healthcare monitoring sensors. This study offers a promising material preparation methodology for stretchable QDs/polymer composites and highlights their potential for advancing flexible and wearable light‐emitting devices.

## Graphical abstract

Intrinsically‐stretchable, heavy‐metal‐free quantum dot color conversion layers are produced via a versatile crosslinking strategy. The color conversion layers exhibit minimal backlight leakage under mechanical strain, support high‐resolution patterning, and integrate seamlessly with micro‐light‐emitting diodes. Their incorporation in haptic‐responsive robotic skin and wearable healthcare sensors highlights their potential for next‐generation stretchable displays.

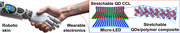

## 1 Introduction

Stretchable displays are vital for next‐generation consumer electronics, driven by innovations in flexible and stretchable device technologies.^[^ ^1^, ^2^, ^3^, ^4^ ^]^ For instance, skin‐like displays serve as essential components for visualizing signals and functioning as light sources in robotic skin and wearable electronics.^[^ ^5^, ^6^, ^7^, ^8^, ^9^, ^10^ ^]^ Beyond simple folding and bending, skin‐like devices must endure diverse mechanical deformations, including stretching, suggesting the importance for advanced stretchable displays. Generally, the skin extension induced by joint movements can reach or exceed ≈30% tensile strain, necessitating stretchable electronics capable of significant mechanical deformation.^[^ ^11^ ^]^ In this context, stretchable light‐emitting diodes (LEDs), featuring intrinsically‐stretchable light‐emitting materials, are considered promising candidates for future display technologies.^[^ ^12^, ^13^, ^14^, ^15^, ^16^, ^17^ ^]^ However, despite recent progress in intrinsically‐stretchable organic‐LEDs (OLEDs) and quantum dot (QD)‐LEDs, these devices face persistent challenges in achieving high efficiency and stability, primarily due to the limited electrical performance of elastomeric components used in stretchable light‐emitting materials.

Micro‐LEDs have emerged as promising light sources for next‐generation displays, offering advantages over traditional LEDs, such as high pixel density, low power consumption, high brightness, fast response times, and long device lifetimes.^[^ ^18^, ^19^, ^20^, ^21^, ^22^ ^]^ Nonetheless, monolithic integration of conventional rigid micro‐LEDs into wearable electronics presents significant challenges due to the rigid mechanical properties of inorganic light‐emitting layers, limiting their multi‐axial mechanical deformations. Conventionally, thereby, structural designs such as island‐bridge configurations have been explored,^[^ ^3^, ^23^, ^24^, ^25^, ^26^ ^]^ where serpentine interconnections that bridge non‐stretchable micro‐LED islands can be stretched and expand the spacings between micro‐LED pixels, leading to stretching of the entire micro‐LED arrays.^[^ ^23^, ^24^, ^25^, ^27^, ^28^, ^29^, ^30^ ^]^ While effective in improving mechanical compliance, this approach results in reduced resolution, decreased brightness, and image distortion during stretching because of the increased spacings between micro‐LED pixels. Moreover, the complicated manufacturing processes required for high‐resolution, full‐color micro‐LED displays—such as precise placement and connection of individual red, green, and blue (RGB) micro‐LED chips—lead to low production yields and high production costs.

Integrating intrinsically‐stretchable color conversion layers (CCLs) onto the structurally‐stretchable micro‐LED arrays provides a practical solution. This strategy enables not only the empty area between micro‐LED chips to be fully filled but also achieves full‐color displays using monochromatic micro‐LED arrays. For this approach to succeed, stretchable CCLs must simultaneously exhibit intrinsic mechanical stretchability, high color purity without backlight leakage, and high‐resolution patternability. Conventional organic phosphor‐ or metal−insulator−metal‐based CCLs have struggled to satisfy these criteria.^[^ ^31^, ^32^ ^]^ QDs have gained attention for CCL applications^[^ ^33^, ^34^, ^35^, ^36^, ^37^ ^]^ due to their high photoluminescence quantum yield (PLQY),^[^ ^38^, ^39^, ^40^, ^41^ ^]^ wide color tunability,^[^ ^42^, ^43^ ^]^ and narrow full width at half maximum (FWHM < 30 nm).^[^ ^38^, ^39^, ^40^, ^41^, ^42^, ^43^, ^44^ ^]^ Despite this promise, developing high‐performance stretchable QD CCLs remains challenging. While QDs/polymer composites have been explored to achieve stretchable CCLs,^[^ ^45^, ^46^, ^47^, ^48^, ^49^ ^]^ the previous systems often suffer from QD aggregations or low QD concentrations, which reduce brightness and color purity due to blue light leakage. Furthermore, issues such as simultaneous achievement of stretchability^[^ ^45^, ^46^, ^47^, ^48^, ^49^, ^50^, ^51^ ^]^ and high‐resolution patterning^[^ ^52^, ^53^, ^54^, ^55^, ^56^ ^]^ (>300 pixels per inch, PPI) persist. Moreover, the reliance on conventional heavy‐metal‐based QDs (e.g., cadmium and lead) in most previous studies^[^ ^36^, ^38^, ^39^, ^40^, ^41^, ^42^, ^43^, ^45^, ^46^, ^47^, ^48^, ^49^, ^50^, ^51^, ^52^, ^53^, ^54^, ^55^, ^56^ ^]^ makes them less desirable for wearable devices or human‐friendly robotic skin applications.

**Figure 1:**
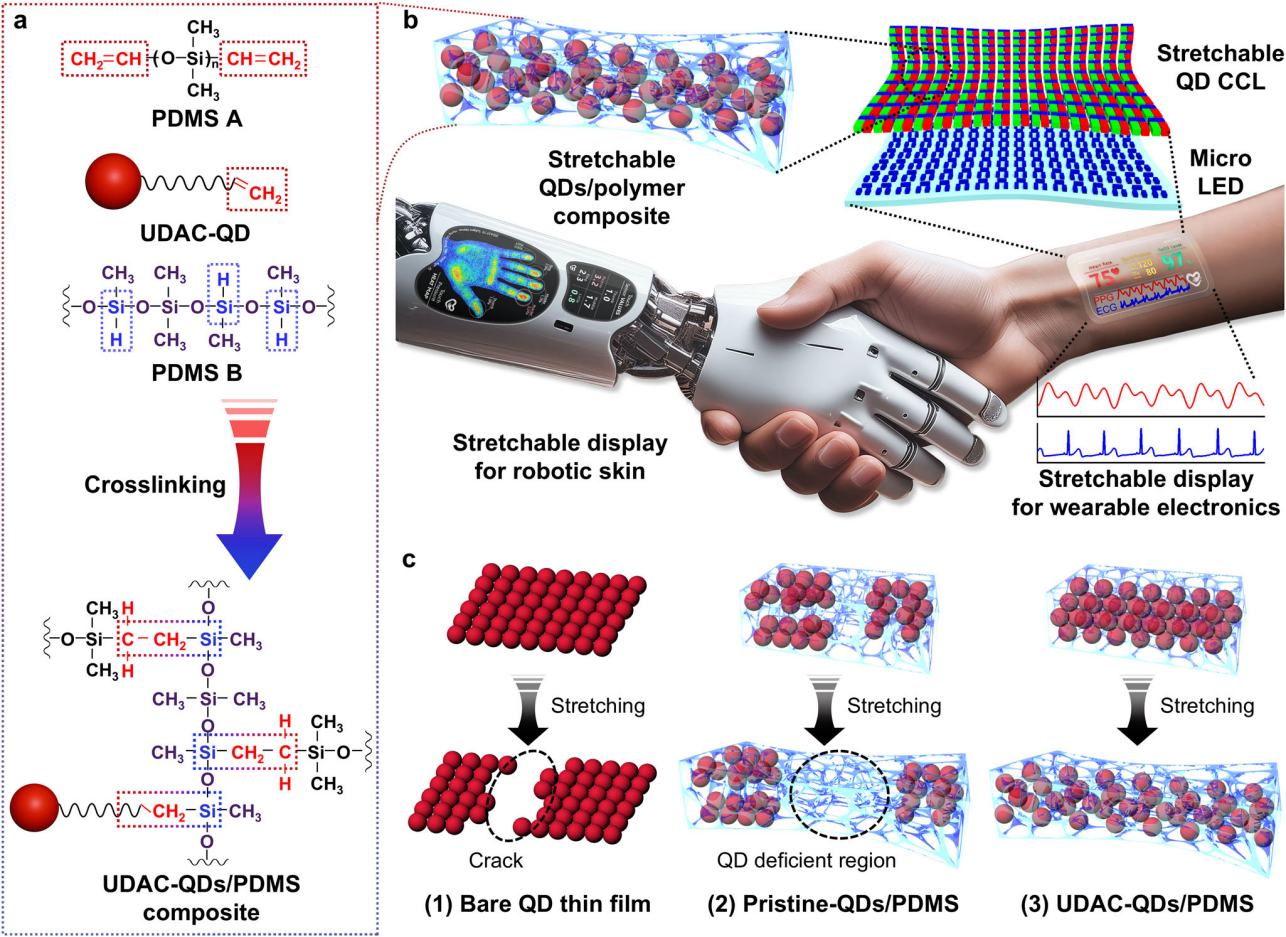
Stretchable QD CCLs for stretchable micro‐LED displays. a) Schematic of UDAC‐QDs/PDMS composite formation through crosslinking between UDAC‐QDs and PDMS, creating highly stretchable QD composites. b) Schematic illustration of stretchable full‐color displays, enabled by integrating stretchable QD CCLs with micro‐LED arrays, designed for applications in robotic skin and wearable electronics. c) Comparison of mechanical behaviors in different QD films under stretching. 1) Conventional QD thin films crack under strain. 2) Simple blending of QDs with stretchable polymers without crosslinking leads to phase separation or low QD loading, creating QD‐deficient regions. 3) Our approach—crosslinking UDAC‐QDs with PDMS—ensures homogeneous QD dispersion with high QD loading, enabling durable and stretchable QD CCLs with uniform performance.

Here, we develop intrinsically‐stretchable and patternable QD CCLs based on heavy‐metal‐free QDs. These CCLs are fabricated by using a controlled crosslinking strategy between QD surface ligands (10‐undecenoic acid, UDAC) and elastomeric polydimethylsiloxane (PDMS), producing composites with exceptional mechanical and optical properties (**Figure** **1**a). This direct crosslinking ensures uniform QD dispersion in the PDMS matrix without phase separation, at high QD loadings, and maintains excellent color conversion with minimal backlight leakage, as demonstrated even at 50% strain. Furthermore, these CCLs support high‐resolution patterning (≈300 PPI), making them suitable for wearable display applications. By integrating these CCLs with monochromatic stretchable micro‐LED arrays, we demonstrate their utility in developing stretchable full‐color micro‐LED displays tailored for robotic skin and wearable electronics applications (Figure 1b).

## 2 Results and Discussion

### 2.1 Preparation of QDs with Crosslinkable Surface Ligands

**Figure 2:**
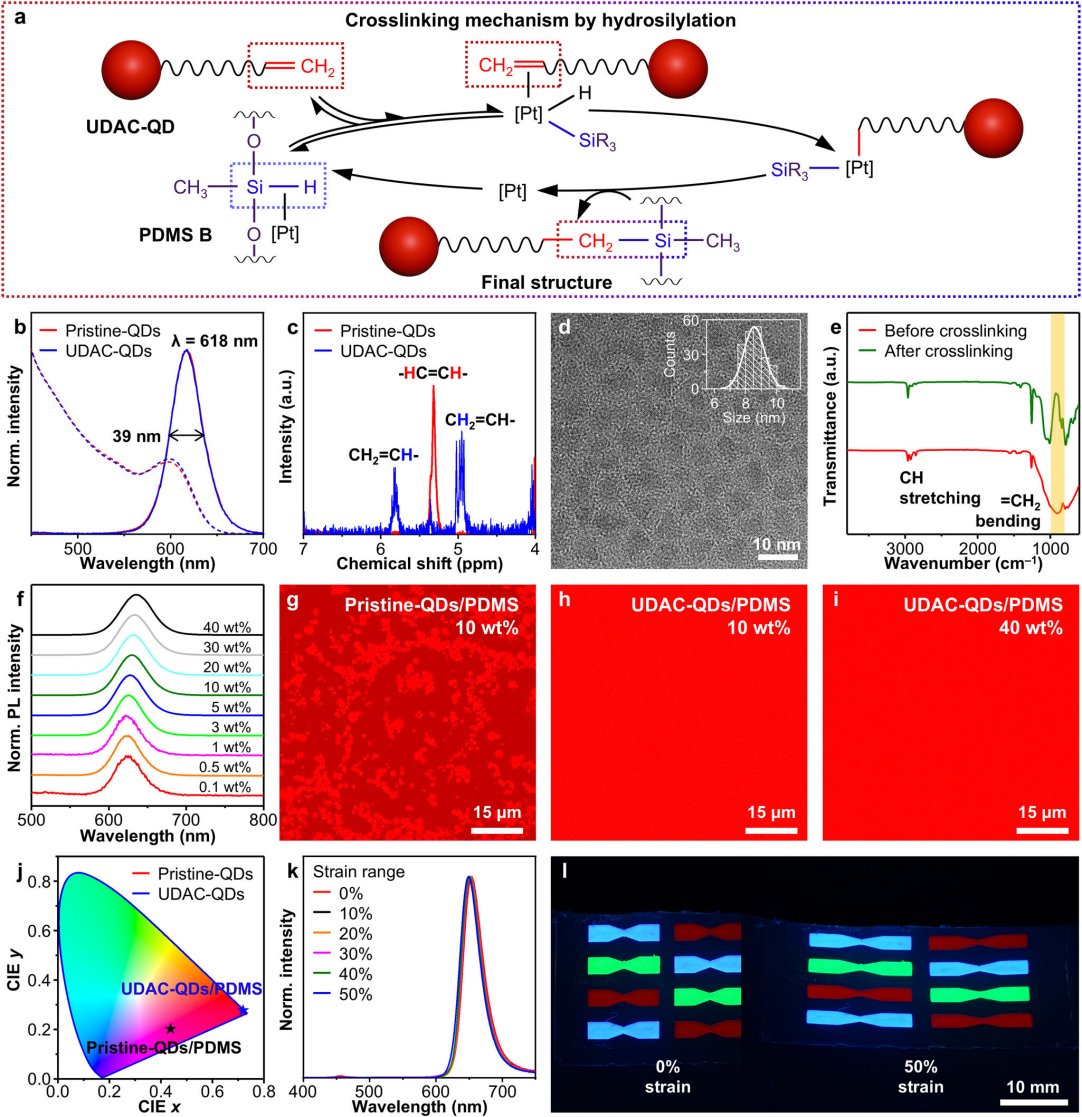
Material design for stretchable QD CCLs. a) Schematic illustration of the crosslinking mechanism between UDAC‐QDs and PDMS via hydrosilylation. b) Absorption (dashed lines) and PL (solid lines) spectra of pristine‐QDs and UDAC‐QDs dispersed in chloroform. c) ^1^H‐NMR spectra of pristine‐QDs and UDAC‐QDs dispersed in chloroform‐*d*. d) TEM image and size distribution (inset) of UDAC‐QDs (*d*_av_ = 8.56 ± 0.66 nm). e) FT‐IR spectra of UDAC‐QDs/PDMS composites before and after curing. f) PL spectra of UDAC‐QDs/PDMS composites with varying QD contents. g–i) Confocal microscope images of (g) pristine‐QDs/PDMS (10 wt.% QDs), (h) UDAC‐QDs/PDMS (10 wt.% QDs), and (i) UDAC‐QDs/PDMS (40 wt.% QDs). j) CIE color coordinates of emissions from micro‐LEDs (λ: 465 nm) employing pristine‐QDs/PDMS and UDAC‐QDs/PDMS CCLs (both 40 wt.% QDs). k) Emission spectra of micro‐LEDs with UDAC‐QDs/PDMS CCLs under varying applied strains. l) Photographs of red‐, green‐, and blue‐emitting UDAC‐QDs/PDMS CCLs, fabricated in a butterfly‐shaped pattern, before and after 50% stretching.

Red‐emitting InP/ZnSe/ZnS QDs were selected as the model system and prepared by colloidal synthesis (see Supporting Information for experimental details) to develop stretchable QD CCLs via the direct crosslinking between QD surface ligands and PDMS (**Figure** **2**a). Unless otherwise specified, “pristine‐QDs” refer to these red‐emitting InP/ZnSe/ZnS QDs without additional surface treatments. They exhibit exceptional optical properties, including strong, narrow red emission at 618 nm (Figure 2b, PLQY >95%, FWHM:39 nm), making them suitable for display applications. These QDs are spherical with an average diameter of 8.66 ± 0.70 nm (Figure S1, Supporting Information), suggesting thick shell structures that contribute to the high stability. However, pristine‐QD films are unsuitable for stretchable QD CCLs due to limited mechanical stretchability (Figure 1c; Figure S2, Supporting Information). While blending QDs with elastomeric polymers is a common approach for creating stretchable composites, this method often leads to local phase separations,^[^ ^45^, ^46^, ^47^, ^48^, ^49^, ^50^, ^51^, ^57^ ^]^ resulting in the formation of QD aggregation and QD‐deficient regions. This causes uneven color conversion and backlight leakage, particularly under stretching (Figure 1c).

To address this challenge, we developed crosslinking chemistry between QDs and elastomeric polymers. For effective CCL applications, the polymer must be optically transparent to minimize light absorption and achieve high display brightness, and exhibit high thermal stability as CCLs can reach temperatures above 180 °C under continuous illumination of micro‐LEDs.^[^ ^58^ ^]^ Consequently, PDMS was chosen as an elastomeric polymer due to its superior stretchability, transparency, and thermal stability (Figure S3, Supporting Information). However, pristine‐QDs lack the functional groups necessary for direct crosslinking with PDMS. To enable this compatibility, we introduced UDACs—carboxylic acids with vinyl terminal groups—as surface ligands for QDs (Figure 2b–d, Experimental Section). These QDs with UDAC surface ligands, which bind to the QD surface via carboxyl groups, are referred to as “UDAC‐QDs”.

Conventionally, PDMS curing occurs through Pt‐catalyzed hydrosilylation reactions, where vinyl groups on PDMS A react with silyl hydride (Si‐H) groups on PDMS B, forming an elastomeric PDMS network (Figure S4, Supporting Information).^[^ ^59^ ^]^ We hypothesize that UDAC‐QDs mimic PDMS A in the curing process due to the terminal vinyl groups of UDAC ligands, enabling their uniform dispersion and firm integration in the viscoelastic composite matrix. Based on this hypothesis, we propose a detailed chemical mechanism for the crosslinking reaction between UDAC‐QDs and PDMS B via conventional hydrosilylation (Figure 2a). In this process, the silyl hydride groups of PDMS B react with the Pt catalysts, transiently forming Si‐Pt‐H intermediates, which facilitate the activation of the terminal vinyl groups on UDAC‐QDs. These activated vinyl groups then undergo insertion into the Si‐Pt‐H intermediate, leading to the formation of Si‐C bonds. Throughout this process, Pt catalysts are released and facilitate repeated catalytic cycles.

To confirm successful UDAC capping on QDs, we employed multiple analytical techniques. Nuclear magnetic resonance (NMR) spectra (Figure 2c; Figure S5, Supporting Information) reveal that pristine‐QDs exhibit a single alkenyl resonance from native oleic acids (5.36 ppm), while UDAC‐QDs display two distinct alkenyl resonances (5.81 and 4.96 ppm) corresponding to vinyl groups.^[^ ^60^, ^61^ ^]^ Thermogravimetric analysis (TGA) further validates UDAC functionalization (Figure S6, Supporting Information), showing the reduced organic content due to the shorter carbon chain of UDAC (C10) compared to the native oleic acids (C18). Importantly, UDAC functionalization preserves the structural and optical properties of QDs. Transmission electron microscopy (TEM) images (Figure 2d; Figure S1, Supporting Information), X‐ray diffraction (Figure S7, Supporting Information), and X‐ray photoelectron spectroscopy analyses (Figure S8, Supporting Information) confirm that the size, shape, crystal structure, and chemical states of QDs remain unchanged after UDAC capping. Additionally, absorption and photoluminescence (PL) spectra of pristine‐ and UDAC‐QDs are nearly identical (Figure 2b), with negligible changes in PLQY (>90%) and carrier lifetime (Figure S9, Supporting Information). These results suggest that UDAC capping does not cause aggregation or introduce defect states of QDs. The high dispersion stability of UDAC‐QDs is also demonstrated by the TEM image of their mixtures with PDMS (Figure S10, Supporting Information).

### 2.2 Fabrication of Stretchable QD CCLs

Stretchable QD CCLs were fabricated by curing UDAC‐QDs with PDMS (Experimental Section). Fourier‐transform infrared (FT‐IR) analysis confirms successful crosslinking, as indicated by the reduction in the = CH_2_ bending signal at 910 cm^−1^ after curing (Figure 2e). Notably, curing is also achievable using only PDMS B and UDAC‐QDs, even without PDMS A, exhibiting a similar trend in FT‐IR analysis (Figure S11, Supporting Information). This provides direct evidence of crosslinking between the vinyl groups on UDAC and the silyl hydride groups on PDMS B, supporting our hypothesis that UDAC‐QDs mimic PDMS A in the curing process. These CCLs exhibit pure red emission, regardless of QD content up to 40 wt.% (Figure 2f). Confocal microscopy reveals the effects of crosslinking on QD distribution in the composites. While pristine‐QDs/PDMS composites show significant QD aggregation due to phase separation across all loadings (Figure 2g; Figure S12, Supporting Information), UDAC‐QDs/PDMS composites display uniform QD dispersion (Figure 2h,i). Furthermore, atomic force microscopy (AFM) analysis confirms the flat and uniform surface morphology (Figure S13, Supporting Information). A QD loading of up to 45 wt.% is suitable for CCL fabrication, maintaining structural integrity, whereas higher loadings (≥50 wt.%) cause QD aggregation, which disrupts crosslinking and leads to partial curing failure (Figure S14, Supporting Information).

The homogeneous and high QD loading enables excellent optical performance. Remarkably, micro‐LEDs (Figure S15, Supporting Information; λ:465 nm) integrated with UDAC‐QDs/PDMS CCLs exhibit pure red emission without blue backlight leakage, even under 50% tensile strain, as confirmed by their consistent color coordinates (Figure 2j; (0.720, 0.274)), emission spectra (Figure 2k), and stable color conversion ratio across various applied strains (Figure S16, Supporting Information). In contrast, pristine‐QDs/PDMS CCLs suffer from significant blue backlight leakage (Figure 2j; Figure S17, Supporting Information), despite a high QD loading of 40 wt.%, which is attributed to QD aggregation. Furthermore, an additional control experiment using traditional rigid QD CCLs based on pristine‐QD films shows significant backlight leakage under tensile strain (Figure S18, Supporting Information) due to limited mechanical stretchability, as evidenced by crack formation in optical microscopy images (Figure S2, Supporting Information).

This crosslinking approach is highly versatile, as demonstrated by its applicability to various QD materials, including green‐emitting InP/ZnSe/ZnS QDs (Figure S19, Supporting Information), red‐, green‐, and blue‐emitting CdSe@ZnS QDs (Figure S20, Supporting Information), and green‐emitting perovskite QDs (Figure S21, Supporting Information). Regardless of the material or emission color, stretchable CCLs with negligible backlight leakage can be achieved, even under 50% stretching (Figure 2l; Figures S22 and S23, Supporting Information), highlighting the general applicability of the crosslinking strategy and its potential for full‐color stretchable displays. Furthermore, UDAC‐QDs/PDMS CCLs exhibit remarkable stability under harsh conditions, including direct water immersion, prolonged heat treatment at 85 °C, and continuous UV irradiation (Figures S24–S26, Supporting Information). This is attributed to the crosslinking network, which significantly enhances QD stability by improving surface passivation.^[^ ^62^, ^63^, ^64^ ^]^

### 2.3 Patterning of Stretchable QD CCLs

**Figure 3:**
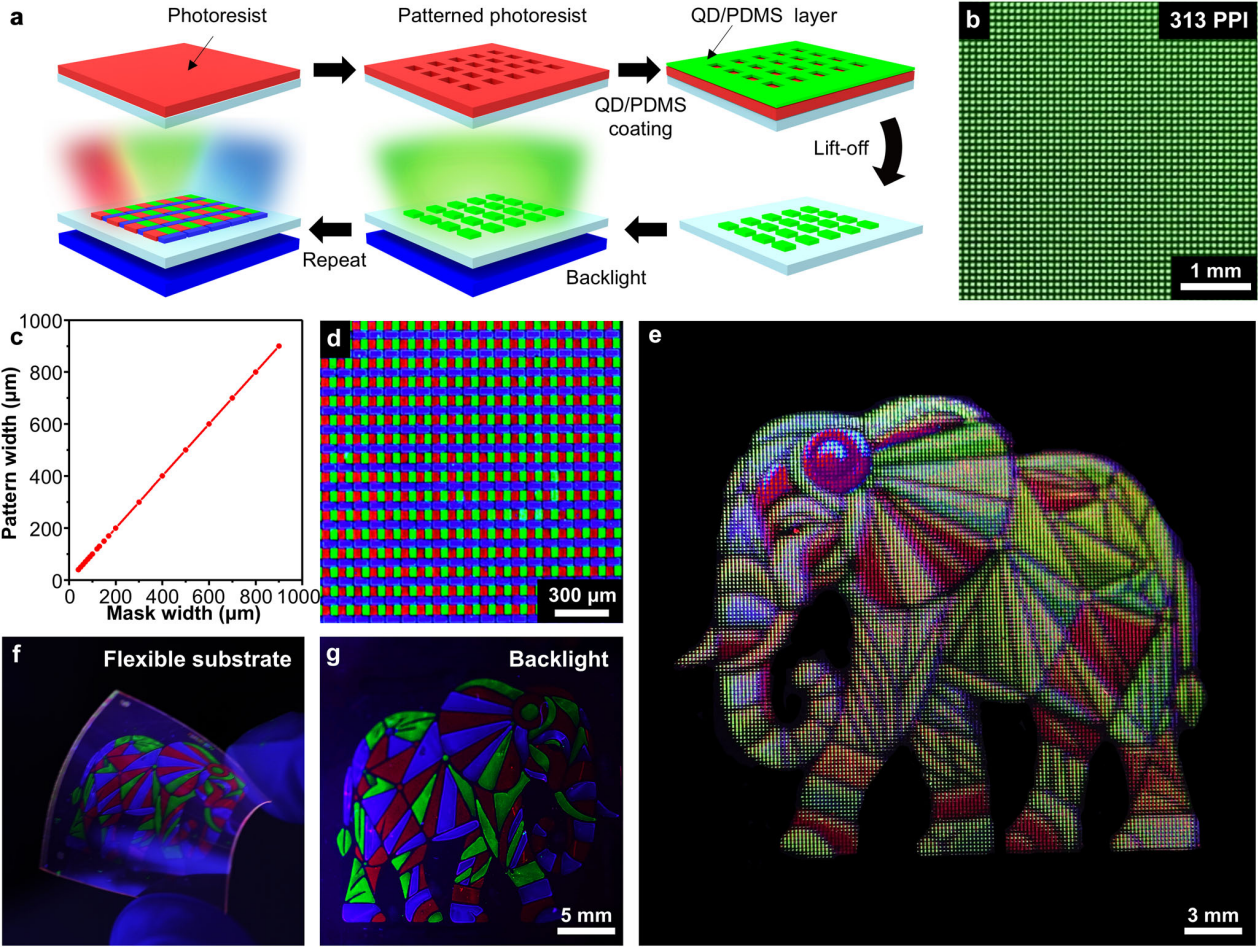
Patterning of stretchable QD CCLs. a) Schematic representation of the lift‐off process used for patterning pixelated RGB QD CCLs. b) PL image of pixelated UDAC‐QDs/PDMS composites with a resolution of 313 PPI. c) Statistical analysis comparing QD CCL pattern widths with corresponding mask widths, ranging from 40 µm to 1 mm. d) PL image of pixelated QD CCLs with RGB sub‐pixels. e) PL image of high‐definition RGB pixelated QD CCLs, displaying an elephant. f) PL image of QD CCL patterns on a flexible PET substrate. g) Color‐converted image achieved by integrating patterned QD CCLs on flexible blue backlight OLEDs.

The UDAC‐QDs/PDMS composites can be patterned and pixelated using the lift‐off process,^[^ ^65^ ^]^ making them suitable for high‐resolution stretchable display applications (**Figure** **3**a, Experimental Section).^[^ ^66^, ^67^ ^]^ First, a positive photoresist was patterned on the target substrate as a mask. The composite solution was then spin‐coated over the photoresist patterns and thermally annealed to induce crosslinking. Unwanted composite regions were removed via the lift‐off process by dissolving the underlying photoresist. This technique enables the fabrication of high‐definition patterns with resolutions up to 313 PPI, which meet the requirements for commercial mobile displays (Figure 3b; Figure S27, Supporting Information). The pattern sizes and shapes precisely match the designed specifications, featuring dimensions ranging from 40 to 900 µm (Figure 3c). Additionally, the pixels exhibit uniform size and shape with no observable microcracks or voids (Figure S28, Supporting Information). Notably, a sufficient thickness for complete color conversion can be achieved for small‐sized pixels (Figure S29, Supporting Information).^[^ ^68^ ^]^ RGB pixels can be fabricated using sequential lift‐off processes (Figure 3d), demonstrating their potential for full‐color displays. Crosslinking of UDAC‐QDs/PDMS composites prevents cross‐contamination between QDs of different colors. By adjusting the relative sizes of RGB sub‐pixels, full‐color images can be generated (Figure 3e). These QD CCL patterns were fabricated on diverse substrates, including glass, silicon, and flexible polyethylene terephthalate (PET, Figure 3f). Strikingly, when integrated on flexible blue OLEDs as a backlight, the patterned CCLs efficiently convert blue light into full‐color images (Figure 3g), indicating their potential for high‐resolution, full‐color stretchable displays.

### 2.4 Stretchable Displays for Robotic Skin

**Figure 4:**
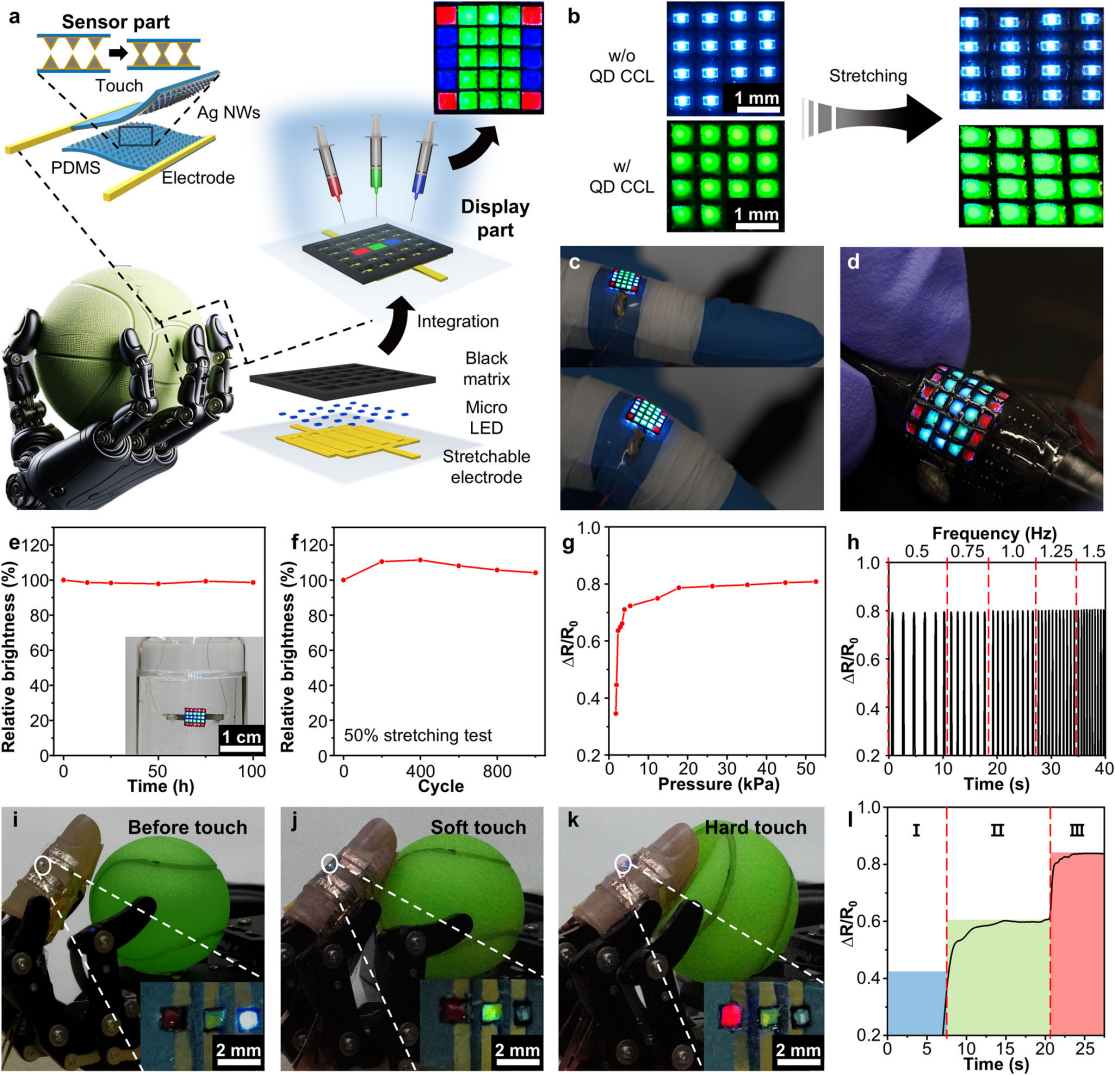
Stretchable displays for robotic skin. a) Schematic illustration of the integration of a resistive touch sensor with a 5 × 5 multicolored stretchable micro‐LED array for robotic skin applications. The top‐right inset shows the photograph of the pixelated 5 × 5 RGB stretchable micro‐LED array, where UDAC‐QDs/PDMS CCLs are deposited into a stretchable black matrix. b) Optical images showing 4 × 4 micro‐LED arrays without (top) and with stretchable QD CCLs (bottom). c,d) Photographs of the stretchable micro‐LED array (c) attached to a finger joint and (d) on a curved surface, demonstrating mechanical flexibility. e) Brightness variation of micro‐LEDs with QD CCLs in an underwater environment. The inset shows the stretchable micro‐LED array submerged in water. f) Brightness stability under repeated 50% stretching cycles. g,h) Resistance changes in the touch sensor under (g) applied pressure, h) diverse frequencies. i–k) Photographs of the robotic skin demonstrating (i) no touch, (j) soft touch, and (k) hard touch responses. Insets show magnified images of the robotic skin, highlighting color changes corresponding to different touch conditions. (l) Resistance changes in the touch sensor attached to the robotic skin under different conditions corresponding to panels (i)–(k): (I) no touch, (II) soft touch, and (III) hard touch.

We applied these stretchable CCLs to fabricate stretchable displays for robotic skin systems.^[^ ^69^ ^]^ **Figure** **4**a illustrates an integrated system comprising the stretchable display and a wearable touch sensor. A 5 × 5 stretchable micro‐LED array was constructed on a stretchable Au electrode array.^[^ ^70^ ^]^ To define pixel boundaries and ensure clear separation, a stretchable carbon‐black‐based black matrix was applied. Multicolor emission was achieved by inkjet‐printing UDAC‐QDs/PDMS CCLs into the patterned regions, as highlighted in the inset image. Conventional stretchable micro‐LED displays often use island‐bridge designs, where rigid micro‐LED “islands” are interconnected by stretchable “bridges”.^[^ ^3^, ^23^, ^24^, ^25^, ^26^ ^]^ Under strain, stretching primarily occurs within these interconnects, reducing the fill factor of the active light‐emitting area and display resolution. For example, traditional designs experience ≈33% drop in fill factor under 50% strain (Figure 4b, top). In contrast, our stretchable QD CCLs cover both micro‐LEDs and interconnect regions, expanding the active area and maintaining fill factor even during stretching deformations (Figure 4b, bottom). The stretchable displays conform to curved surfaces like finger joints while ensuring stable optical performance (Figure 4c,d). Moreover, QD CCLs protect the micro‐LED arrays, enabling reliable operation for 100 h underwater (Figure 4e) and after repeated stretching cycles with 50% strain (Figure 4f).

We developed functional robotic skins capable of real‐time force visualization via tactile recognition^[^ ^71^, ^72^, ^73^ ^]^ by integrating the stretchable micro‐LED display with a wearable resistive touch sensor. The upper‐left section of Figure 4a schematically illustrates the structure and operating principle of the resistive touch sensor, which features pyramid‐shaped microstructures composed of silver nanowire (Ag NW)‐embedded PDMS (Figure S30, Supporting Information). When pressure is applied, these pyramid structures deform, increasing the contact area and reducing resistance. This resistance change is proportional to the applied pressure, enabling precise pressure sensing. To ensure effective tactile perception, we designed the pressure response range of our robotic skin to mimic that of human skin, covering 0 to tens of kPa (Figure 4g).^[^ ^74^ ^]^ The tactile sensor maintains stable resistance across varying frequencies, demonstrating reliability in dynamic conditions (Figure 4h). The applied pressure on the tactile sensor is simultaneously visualized through the stretchable micro‐LED display integrated into the robotic skin. In the absence of contact, the display emits blue light (Figure 4i). When the robotic skin gently grasps a plastic ball with an appropriate pressure level (<20 kPa), the display shifts to green (Figure 4j). However, excessive pressure that deforms the ball triggers red light emission, signaling overexertion (Figure 4k). The corresponding real‐time pressure data for this demonstration is presented in Figure 4l. This intuitive real‐time feedback enhances precision and interactivity in robotic manipulation tasks, demonstrating the potential of stretchable sensor‐integrated robotic skins.

### 2.5 Stretchable Sensors for Wearable Healthcare Monitoring

**Figure 5:**
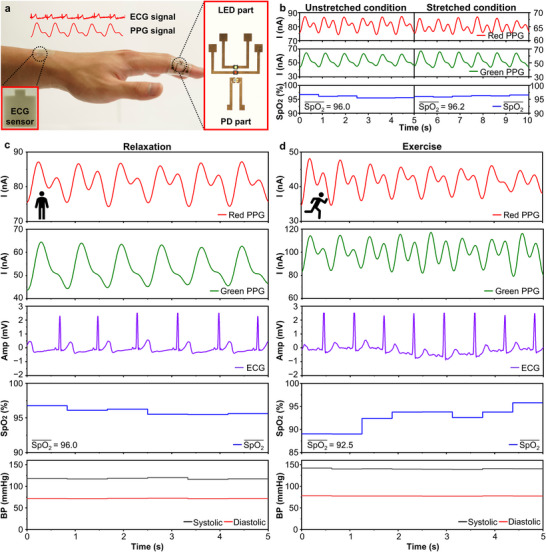
Stretchable PPG sensors for real‐time health monitoring. a) Optical image of the wearable healthcare monitoring system. Insets show magnified views of the stretchable ECG (left) and PPG (right) sensors. b) PPG signals and SpO_2_ values measured using red and green emissions under unstretched and 50% stretched conditions. c,d) Physiological signals, including PPG, ECG, SpO_2_ levels, and blood pressure, monitored under (c) relaxation and (d) exercise.

Finally, stretchable QD CCLs were employed to develop stretchable photoplethysmogram (PPG) sensors for real‐time healthcare monitoring.^[^ ^75^, ^76^, ^77^, ^78^, ^79^, ^80^ ^]^ **Figure** **5**a presents skin‐attachable biosensors, including a stretchable electrocardiogram (ECG) sensor (left inset) and a stretchable PPG sensor (right inset). The stretchable PPG sensor, operating in the reflection mode, is constructed by integrating blue micro‐LEDs and a micro‐photodetector (PD) onto stretchable micro‐cracked Au electrodes. Red and green QD CCLs were integrated with the LEDs for red and green emissions, respectively. Variations in blood volume caused by heartbeats alter the reflected light intensity, which is detected by the PDs and converted into electrical signals to generate PPG waveforms. By analyzing these waveforms from two distinct light colors, the sensor extracts key physiological data, including heart rate and blood oxygen saturation (SpO_2_, see Supporting Information).^[^ ^81^, ^82^, ^83^ ^]^

Stretchable PPG sensors effectively monitor vital signs during diverse activities by capturing dynamic physiological changes. Their performance under mechanical deformation was evaluated by comparing PPG signals and SpO_2_ levels in unstretched and 50% stretched states. Because of their conformal skin contact on the fingertip, the sensors presented consistent heart rate (≈72 beats per minute, bpm) and SpO_2_ levels (≈96.0%), regardless of 50% stretching, suggesting their robustness and reliability during various activities of users (Figure 5b). Notably, simultaneous ECG and PPG measurements allow for the extraction of multiple vital signs (see Supporting Information),^[^ ^84^, ^85^, ^86^ ^]^ such as pulse, SpO_2_ levels, and blood pressure (Figure 5c,d). The ECG signal was obtained by attaching Ag NW‐based stretchable electrodes to the skin and measuring the potential difference between the electrodes (Figure S31, Supporting Information). During exercise, significant physiological changes were observed compared to the relaxed states, including an increase in heart rate from 72 to 96 bpm observed in both PPG and ECG signals, a decrease in SpO_2_ levels from 96.0% to 92.5%, and a rise in blood pressure, with systolic pressure from 120.0 to 142.0 mmHg and diastolic pressure from 72.5 to 78.0 mmHg. The conformal skin contact minimizes motion artifacts, ensuring accurate signal acquisition during diverse activities. These results highlight the potential of stretchable PPG sensors as key components for advanced wearable healthcare monitoring systems.

## 3 Conclusion

This study demonstrates the successful development of intrinsically‐stretchable and patternable QD CCLs for stretchable micro‐LED displays, which can be potentially applied to wearable electronics and robotic skin. Using the versatile crosslinking strategy between QDs and elastomer, we achieve homogenous and high QD loading, excellent optical properties, and mechanical durability, overcoming key limitations of previous QDs/polymer composite systems. The stretchable UDAC‐QDs/PDMS CCLs exhibit exceptional performance, including high‐resolution patterning (≈300 PPI) and stable color conversion capabilities without backlight leakage, even under 50% applied strain. Their integration with micro‐LED displays enables robust, full‐color stretchable displays. We further showcase their versatility through practical applications, such as robotic skins for dynamic force visualization and stretchable PPG sensors for real‐time health monitoring. Our results demonstrate that the stretchable QD CCLs can be an effective solution for the development of next‐generation high‐resolution stretchable micro‐LED displays.

## 4 Experimental Section

### Synthesis of Colloidal QDs

QDs were prepared by colloidal synthesis, and the detailed synthesis procedures are described in the Supporting Information. The chemicals used in this study are listed in the Supporting Information.

### Functionalization of QDs with UDAC

The ligand exchange process was performed for UDAC functionalization of QDs, which were initially passivated with native surface ligands from QD synthesis. UDAC was heated at 120 °C under vacuum with stirring for 2 h to eliminate moisture and unwanted gases. Subsequently, 1.0 mL of QD solution (20 mg mL^−1^ in hexane) was mixed with 40.0 µL of UDAC. The mixture was stirred under an argon atmosphere at 25 °C for 24 h. To remove excess ligands, the mixture underwent two consecutive centrifugation steps using 1‐propanol. The resulting UDAC‐QDs were re‐dispersed in chloroform (10 mg mL^−1^) for further applications.

### Fabrication of QDs/PDMS CCLs

To fabricate QDs/PDMS CCLs, QDs dispersed in chloroform (10 mg mL^−1^) were mixed with the PDMS compound solution (i.e., PDMS A: PDMS B = 40:1 in chloroform) at a specified ratio. The mixture was dried under vacuum at 40 °C for 1 h to evaporate the chloroform. Subsequently, the mixture was deposited onto various molds (e.g., patterned PDMS, black matrix, photoresists, etc.) using the blade coating technique. The thickness of CCLs could be controlled from a few micrometers to ≈100 µm. Finally, the composites were cured under vacuum at 150 °C over 2 h.

### QD Patterning Via Lift‐Off Process

Photoresist (AZ 4620) patterns were fabricated on the target substrate (e.g., glass, Si wafer, and PET) to serve as a mask for the lift‐off process. The UDAC‐QDs/PDMS composite was spin‐coated onto the photoresist patterns and subsequently cured on a hot plate at 150 °C for 20 min. The lift‐off process was executed by immersing the substrates in acetone, effectively dissolving the underlying photoresist and removing the undesired QDs/PDMS parts. The resulting patterns were then thoroughly rinsed with isopropyl alcohol and dried using nitrogen gas.

### Fabrication of the Stretchable 5 × 5 RGB Micro‐LED Array

A stretchable Au electrode was fabricated on a 50 µm‐thick SEBS substrate by depositing 60 nm‐thick Au at a controlled rate of 0.1 Å s^−1^. Blue micro‐LEDs (λ: 465 nm, Kingbright) were precisely aligned and positioned onto the Au electrodes using conductive epoxy, which was cured by annealing at 80 °C for 1 h in an oven. A stretchable black matrix was manufactured using a composite of carbon black, Ecoflex, and PDMS in a weight ratio of 1:10:3. The composite was subsequently cured and laser‐cut to create the desired pixels. The black matrix was integrated with the micro‐LED array using a commercial adhesive. RGB UDAC‐QD/PDMS composites were inkjet‐printed into each pixel cavity using a micro‐syringe and annealed in a vacuum oven at 80 °C for 3 h.

### Device Characterization

The emission spectra of micro‐LEDs with QD CCLs were measured using a Konica Minolta CS‐2000 spectroradiometer. The resistance of the touch sensor was measured with a Fluke True root mean square (RMS) multimeter. The PPG data was acquired using a Stanford Research Systems SR‐570 low noise current preamplifier and data acquisition (DAQ) system. The collected PPG signal was filtered with a fast Fourier transform filter. The ECG data were measured with a PhysioLab iDAQ‐400 biosignal recorder. All experiments involving biosignal monitoring sensors, including PPG and ECG signal measurements, were conducted with healthy adult participants in compliance with the protocol approved by the Institutional Review Board (IRB) of the Ulsan National Institute of Science and Technology (Approval No. UNISTIRB‐23‐028‐A). Prior to participation, all individuals provided written informed consent.

## Conflict of Interest

The authors declare no conflict of interest.

## Supporting information



Supporting Information

## Data Availability

The data that support the findings of this study are available in the supplementary material of this article.
