# Supplementary material for: Intrinsically‐Stretchable and Patternable Quantum Dot Color Conversion Layers for Stretchable Displays in Robotic Skin and Wearable Electronics
Source: Adv Mater. 2025 May 6;37(32):2420633. doi: 10.1002/adma.202420633 (PMC12355528; doi:10.1002/adma.202420633)
Supplement: Supplementary file 1 — Supporting Information [file ADMA-37-2420633-s001.pdf]

ADVANCED MATERIALS

Supporting Information

for *Adv. Mater.*, DOI 10.1002/adma.202420633

Intrinsically-Stretchable and Patternable Quantum Dot Color Conversion Layers for Stretchable Displays in Robotic Skin and Wearable Electronics

Kiwook Kim, Dong Ryong Kim, Dohyeon Kim, Hyeon Hwa Song, Seungmin Lee, Yonghoon Choi, Kyunghoon Lee, Gwang Heon Lee, Jinhee Lee, Hye Hyun Kim, Eonhyoung Ahn, Jae Hong Jang, Yaewon Kim, Hyo Cheol Lee, Yunho Kim, Soo Ik Park, Jisu Yoo, Youngsik Lee, Jongnam Park, Dae-Hyeong Kim*, Moon Kee Choi* and Jiwoong Yang**

Supporting Information

Intrinsically-Stretchable and Patternable Quantum Dot Color Conversion Layers for Stretchable Displays in Robotic Skin and Wearable Electronics

Kiwook Kim,[†] Dong Ryong Kim,[†] Dohyeon Kim, Hyeon Hwa Song, Seungmin Lee, Yonghoon Choi, Kyunghoon Lee, Gwang Heon Lee, Jinhee Lee, Hye Hyun Kim, Eonhyoung Ahn, Jae Hong Jang, Yaewon Kim, Hyo Cheol Lee, Yunho Kim, Soo Ik Park, Jisu Yoo, Youngsik Lee, Jongnam Park, Dae-Hyeong Kim,* Moon Kee Choi,* and Jiwoong Yang**

K. Kim, H. H. Song, K. Lee, J. Lee, E. Ahn, H. C. Lee, S. I. Park, Prof. J. Yang
Department of Energy Science and Engineering, Daegu Gyeongbuk Institute of Science and Technology (DGIST), Daegu 42988, Republic of Korea
*E-mail: jiwoongyang@dgist.ac.kr

D. R. Kim, G. H. Lee, Y. Kim, Y. Kim, Prof. M. K. Choi
Graduate School of Semiconductor Materials and Devices Engineering, Center for Future Semiconductor Technology (FUST), Ulsan National Institute of Science and Technology (UNIST), Ulsan 44919, Republic of Korea
*E-mail: mkchoi@unist.ac.kr

D. Kim, Y. Lee, Prof. D.-H. Kim
School of Chemical and Biological Engineering, Institute of Chemical Processes, Seoul National University, Seoul 08826, Republic of Korea
*E-mail: dkim98@snu.ac.kr

D. Kim, Y. Lee, Prof. D.-H. Kim
Center for Nanoparticle Research, Institute for Basic Science (IBS), Seoul 08826, Republic of Korea

S. Lee, H. H. Kim, J. H. Jang, J. Yoo, Prof. M. K. Choi
Department of Materials Science and Engineering, Ulsan National Institute of Science and Technology (UNIST), Ulsan 44919, Republic of Korea

Y. Choi, Prof. J. Park
School of Energy and Chemical Engineering, Ulsan National Institute of Science and Technology (UNIST), Ulsan 44919, Republic of Korea
*E-mail: jnpark@unist.ac.kr

H. C. Lee
Department of Chemistry, Hong Kong University of Science and Technology (HKUST), Kowloon 999077, Hong Kong SAR, Hong Kong

Prof. J. Park
Department of Biomedical Engineering, Ulsan National Institute of Science and Technology (UNIST), Ulsan 44919, Republic of Korea

Prof. J. Yang
Energy Science and Engineering Research Center, Daegu Gyeongbuk Institute of Science and Technology (DGIST), Daegu 42988, Republic of Korea

[†]These authors contributed equally to this work.

Keywords: Stretchable display, Stretchable color conversion layer, Quantum dot, Wearable electronics, Robotic skin

1. Supporting Methods

Materials: Indium(III) acetate ($In(Ac)_3$, 99.99% trace metal basis), oleic acid (OA, 90% technical grade), lauric acid (LA, 98%), 1-octadecene (ODE, 90% technical grade), 10-undecenoic acid (UDAC, 98%), zinc acetate ($Zn(Ac)_2$, 99.999% trace metal basis), selenium (Se, 99.99% trace metal basis), sulfur (S, 99.5% trace metals basis), 1-dodecanethiol (DDT, >98%), trioctylamine (TOA, 98%), trioctylphosphine (TOP, 97%), zinc acetylacetonate ($Zn(acac)_2$, for synthesis), zinc oxide (ZnO, 99.99%), cadmium acetate ($Cd(Ac)_2$, 99.995%), toluene (anhydrous, 99.8%), lead(II) bromide ($PbBr_2$, 99.998% trace metals basis), dodecanoic acid (98%), chloroform (anhydrous, 98.5%), hydrofluoric acid (HF, 48%), carbon black, and octadecyltrichlorosilane (ODTS, >90%) were purchased from Sigma-Aldrich. 1-Propanol (99.5%), isopropyl alcohol (99.5%), *n*-hexane (96%), and methyl acetate (anhydrous, 99.5%) were purchased from Samchun Chemical. Tris(triemethylsilyl)phosphine (TMS_3P, 99.0%) was purchased from SK Chemicals. Indium(III) acetylacetonate ($In(acac)_3$, 99%) and didodecyl dimethyl ammonium bromide (DDAB, >98.0%) were purchased from TCI Chemical. Blue micro-light-emitting diode (LED, λ: 465 nm) chips were purchased from Kingbright. Flexible blue organic LEDs were purchased from Obang. Styrene-ethylene-butylene-styrene (SEBS) elastomer was purchased from Asahi Kasei. Ecoflex was purchased from Smooth-On. Polydimethylsiloxane (PDMS) was purchased from Dow Corning. Silver nanowires (Ag NWs, 1 wt% in isopropanol) were purchased from SG Flexio Co., Ltd.

Synthesis of red-emitting InP/ZnSe/ZnS quantum dots (QDs): For the preparation of indium laurate ($In(LA)_3$) complexes, a mixture containing 20.0 mmol of $In(Ac)_3$ and 60.0 mmol of dodecanoic acid was heated under vacuum at 160 °C for 3 h. Subsequently, 130.0 mL of ODE was added, and the mixture was further heated under vacuum at 120 °C for 3 h.

For the preparation of zinc oleate, a mixture containing 72.0 mmol of $Zn(Ac)_2$ and 48.0 mL of OA was heated under vacuum at 160 °C for 3 h. Subsequently, 100.0 mL of TOA was added, and the mixture was further heated under vacuum at 120 °C for 3 h.

For the preparation of In-Zn-P complex, a mixture containing 1.0 mmol of $In(acac)_3$, 0.5 mmol of $Zn(acac)_2$, 4.0 mmol of OA, and 10.0 mL of ODE was heated under vacuum at 120 °C for 2 h. Subsequently, at room temperature, 1.19 mL of TMS_3P solution (10 vol% in TOP) was injected and allowed to react for 1 h.

For the synthesis of InP core QDs, a mixture containing 0.32 mmol of $In(LA)_3$, 0.32 mmol of zinc oleate, and 1.0 mL of ODE was heated under vacuum at 120 °C for 2 h. Subsequently, 0.8 mL of TMS_3P solution (10 vol% in TOP) was injected at 110 °C and allowed to react for

20 min. Subsequently, the mixture was heated to 265 °C and the reaction was carried out for 45 min. Following this, the temperature was raised to 300 °C, and the In-Zn-P complex solution was slowly injected until the 1st excitonic peak of InP core QDs reached 580 nm.

For the synthesis of InP/ZnSe/ZnS QDs, a mixture containing 5.0 mL of the InP core QD solution, 3.0 mL of zinc oleate, 6.16 g of TOA, 1.6 mL of TOP, and 0.15 mL of TOP-Se (2 M) was heated under vacuum at 120 °C for 2 h. Then, 0.05 mL of HF solution (10 wt% in acetone) was injected at 180 °C under an argon atmosphere and allowed to react for 30 min. Subsequently, the mixture was heated to 340 °C, and 0.15 mL of TOP-Se (1 M) was introduced for ZnSe shell growth. After 30 min, 3.0 mL of the zinc oleate complex solution was injected, and the reaction temperature was lowered to 320 °C. To grow the ZnS shells, 0.15 mL of TOP-S (1 M) was injected. The final products were purified by standard centrifugation with acetone (7,500 rpm for 5 min), repeated three times. The precipitates were dissolved in hexane for subsequent experiments.

Synthesis of green-emitting InP/ZnSe/ZnS QDs: A mixture containing 0.3 mmol of $In(Ac)_3$, 10.0 mL of ODE, and 0.415 mL of OA was heated under vacuum at 120 °C for 2 h. Subsequently, 1.0 mL of the TMS_3P solution was injected at room temperature. Then, the mixture was heated to 300 °C for 20 min, after which 0.2 mL of TOP-Se (1 M) was slowly introduced for the ZnSe shell growth. For the additional shell growth, a mixture containing 1.5 mL InP QD solution, 2.8 mmol $Zn(Ac)_2$, 6.0 mL of TOA, and 0.635 mL of OA was heated under vacuum at 120 °C for 2 h. Subsequently, 0.5 mL of TOP-Se (1 M) and 0.1 mL of TOP-S (1 M) were injected under an argon atmosphere. The reaction mixture was heated to 320 °C for 1 h. This process was repeated once more without adding TOP-Se. The final products were purified by standard centrifugation with 1-propanol (7,500 rpm for 5 min), repeated three times. The precipitates were dissolved in hexane for subsequent experiments.

Synthesis of red-, green-, and blue-emitting CdSe@ZnS QDs: Red-, green-, and blue-emitting CdSe@ZnS QDs were synthesized using a method adapted from a previous report.[S1]

Synthesis of green-emitting $CsPbBr_3$ perovskite QDs with green emission: Green-emitting $CsPbBr_3$ perovskite QDs were synthesized using a method adapted from a previous report.[S2]

Fabrication of the stretchable touch sensor: Ag NWs dispersed in isopropanol (1 wt%) were spray-coated onto a pre-cleaned, pyramid-shaped Si master mold. The PDMS solution was then

spin-coated over the Ag NW-coated mold and cured at 80 °C for 2 h in an oven. After curing, the Ag NW-embedded PDMS layer was carefully peeled off from the mold. Two pyramid-structured PDMS layers were subsequently stacked to fabricate a stretchable resistive touch sensor.

Fabrication of the electrocardiogram (ECG) sensors: Ag NWs dispersed in isopropanol (1 wt%) were spray-coated onto an ODTS-treated Si substrate using a shadow mask with the desired pattern. Then, polyurethane acrylate (PUA) was cast onto the patterned Ag NWs and cured under UV light. The cured PUA film was carefully peeled off from the substrate, completing the ECG sensor fabrication. The deformability of our ECG sensor ensures precise data detection by conformally attaching to the skin. ECG signals were obtained by measuring the potential difference between electrodes using a Physiolab iDAQ-400 biosignal recorder.

Fabrication of the photoplethysmogram (PPG) sensors: Stretchable Au electrodes were deposited on a 50-μm-thick SEBS substrate using thermal evaporation at a controlled rate of 0.1 Å/s. Photodetectors and micro-LED chips were integrated onto the Au electrodes using conductive epoxy, followed by annealing at 80 °C for 1 h. Subsequently, red and green UDAC-QDs/PDMS composites were deposited via inkjet printing. To enhance mechanical stability and biocompatibility, a thin stretchable PDMS encapsulation layer was applied.

Material characterization: Transmission electron microscopy (TEM), scanning transmission electron microscopy (STEM), and energy-dispersive X-ray spectroscopy (EDS) images were obtained using a FEI Tecnai G2 F20 TWIN TMP microscope operated at 200 kV. EDS mapping images were acquired with a Horiba EX-200. Scanning electron microscopy (SEM) images were captured using a Hitachi SU-8230. ^{1}H-nuclear magnetic resonance (NMR) spectra were measured using a 600 MHz Bruker BioSpin AG system. Thermogravimetric analysis (TGA) was conducted using a Thermo Plus EVO Rigaku TG8120. X-Ray diffraction (XRD) patterns were recorded using a Rigaku Miniflex 600 X-ray diffractometer with Cu Kα radiation (λ=0.15406 nm) over a 2 theta range of 20–80°. X-ray photoelectron spectroscopy (XPS) spectra were measured with a Thermo scientific ESCALAB 250Xi system using Al Kα radiation (1486.6 eV). Absorption spectra were obtained with a Cary 5000 UV-Vis-NIR spectrophotometer. Photoluminescence (PL) and time-resolved PL decay spectra were recorded using a Horiba Fluoromax-4 spectrophotometer. Fourier-transform infrared (FT-IR) spectra were acquired using an Agilent Cary 660 FTIR spectrometer. Confocal microscope images were

captured using a Carl Zeiss LSM900. Atomic force microscopy (AFM) images were acquired by Oxford Instrument MFP-3D Origin™ AFM. Optical microscopy images were acquired by OLYMPUS BX53M.

2. Supporting Notes

Measurement of oxygen saturation (SpO_2) levels: SpO_2 levels were measured using PPG sensors that emit red and green light, utilizing the distinct optical absorption properties of oxygenated hemoglobin (HbO_2) and deoxygenated hemoglobin (Hb). Red light penetrates deeper into tissue and is predominantly absorbed by Hb, while green light exhibits higher sensitivity to blood flow dynamics at shallower tissue depths. By analyzing the intensity variations in the reflected light, the relative concentrations of HbO_2 and Hb were determined. The SpO_2 calculation is based on the absorbance ratio, expressed as:

$$SpO_2 = A - B \times R \qquad \text{(Eq. S1)}$$

where A and B are empirically determined constants and R is derived from the PPG data using the equation:

$$R = \frac{AC_{red}/DC_{red}}{AC_{green}/DC_{green}} \qquad \text{(Eq. S2)}$$

where AC and DC represent the alternating current and direct current components of the red and green light signals, respectively. These components were extracted from the PPG waveforms, as illustrated in **Supporting Note Figure 1**.

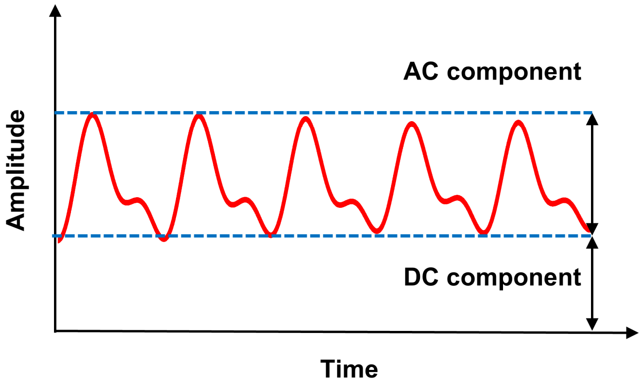

Supporting Note Figure 1. Illustration of the AC and DC components of the PPG signals.

Measurement of blood pressure using pulse transit time (PTT): PTT refers to the time interval required for a pressure wave, generated by a heartbeat, to travel from the heart to peripheral blood vessels. It quantifies the velocity of the pressure waveform as it propagates from the aorta through the arterial system to peripheral sites. As shown in **Supporting Note Figure 2**, the PTT value was calculated using ECG and PPG signals, based on the following equation:

$$PTT = T_{PPG} - T_{ECG} \qquad \text{(Eq. S3)}$$

where T_{PPG} represents the time of PPG signal detection at the peripheral site, and T_{ECG} corresponds to the R-wave peak time in the ECG signal. Blood pressure was calculated using the PTT value, based on the following equation:[S3]

$$\text{Blood Pressure} = a - b \times \text{PTT} \qquad \text{(Eq. S4)}$$

where *a* and *b* are empirically determined constants.

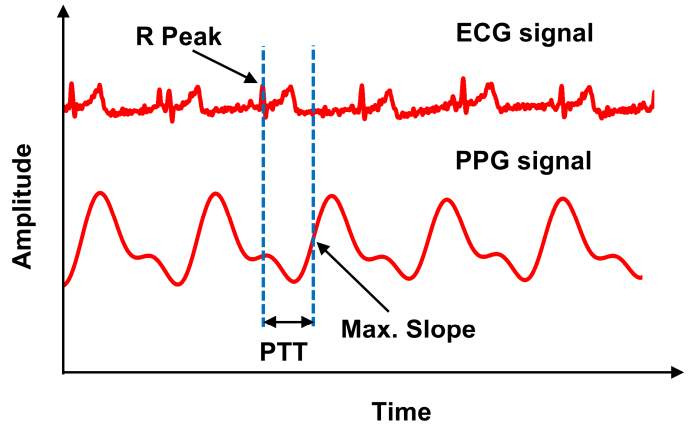

Supporting Note Figure 2. Illustration of PTT measurements derived from ECG and PPG signals.

3. Supporting Figures

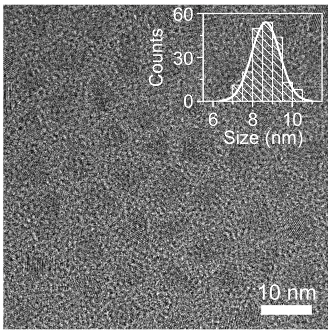

Figure S1. TEM image of pristine-red-emitting InP/ZnSe/ZnS QDs. The inset shows the size distribution of the QDs (average diameter, d_{av} = 8.66 ± 0.70 nm; n = 200).

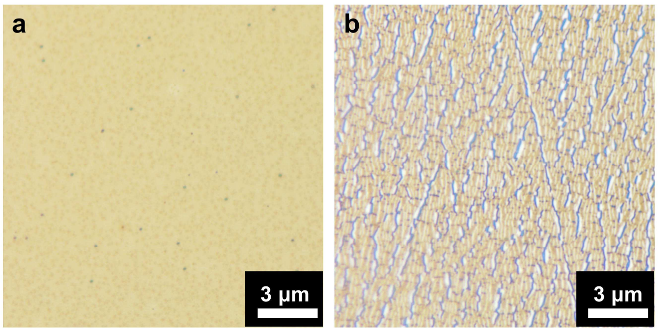

Figure S2. Optical microscopy analysis of pristine-QD films. a,b) Optical microscopy images of the pristine-QD films (a) before and (b) after 50% stretching.

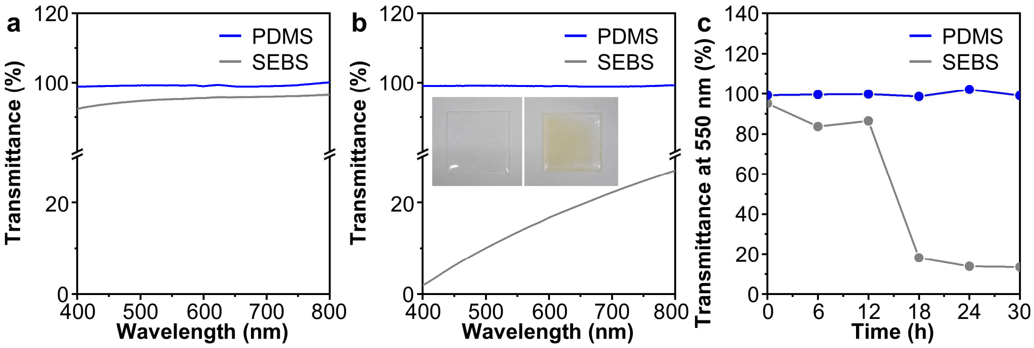

Figure S3. Comparison between PDMS and SEBS. a,b) Transmittance spectra of PDMS and SEBS (a) before and (b) after heating at 180 °C for 30 h. The inset in panel (b) shows photograph images of each sample. c) Transparency of PDMS and SEBS at 550 nm as a function of the heating time.

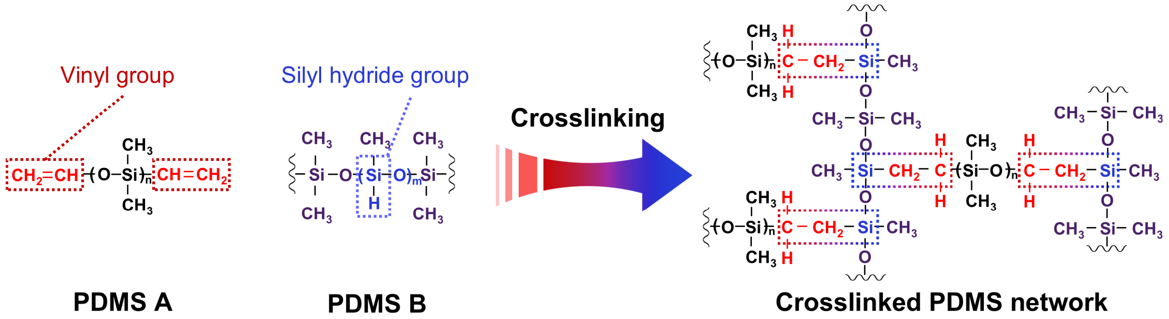

Figure S4. Schematic illustration of conventional curing process of PDMS. PDMS A consists of a base silicone polymer with methyl siloxane repeating units and is functionalized by vinyl group. PDMS B typically acts as the crosslinker in the curing process and contains functional groups such as silyl hydride (Si-H) group. In the conventional curing process, the vinyl groups on PDMS A react with the silyl hydride groups on PDMS B, forming a solid, crosslinked PDMS network.

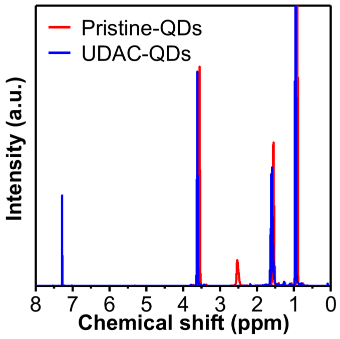

Figure S5. ^{1}H-NMR spectra of pristine-QDs and UDAC-QDs.

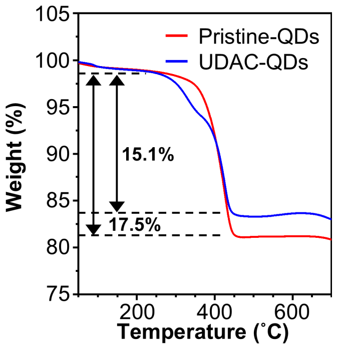

Figure S6. TGA curves of pristine-QDs and UDAC-QDs.

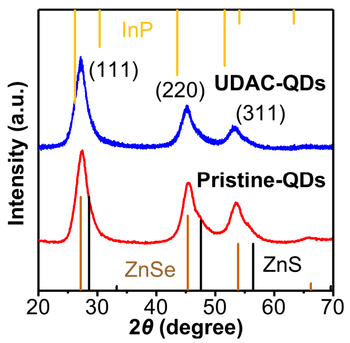

Figure S7. XRD patterns of pristine-QDs and UDAC-QDs. The reference XRD data for bulk InP (yellow, JCPDS No.: 10-0216), ZnSe (brown, JCPDS No.: 01-0690), and ZnS (black, JCPDS No.: 01-0792) crystals are shown together for comparison.

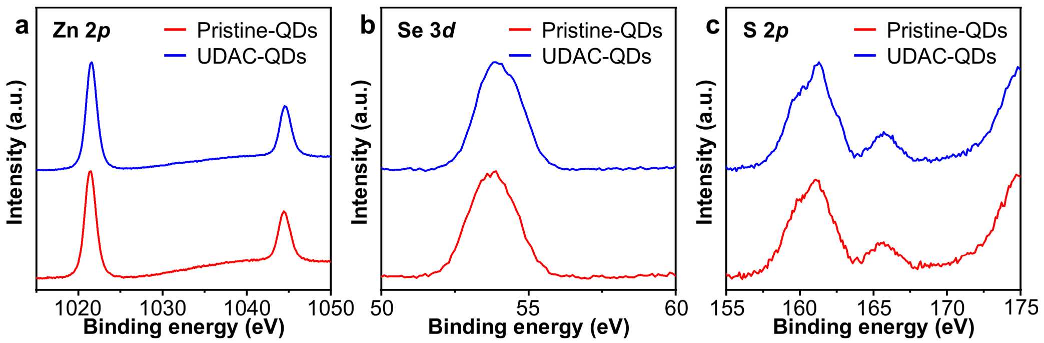

Figure S8. XPS analysis of pristine-QDs and UDAC-QDs. a–c) XPS spectra for (a) Zn-2*p*, (b) Se-3*d*, and (c) S-2*p* regions of pristine-QDs and UDAC-QDs.

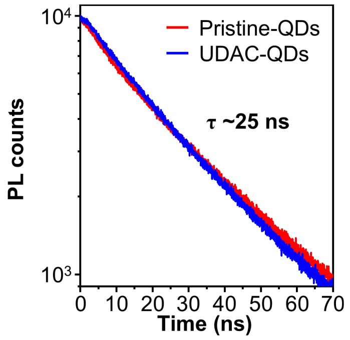

Figure S9. Time-resolved PL decay spectra of pristine-QDs and UDAC-QDs.

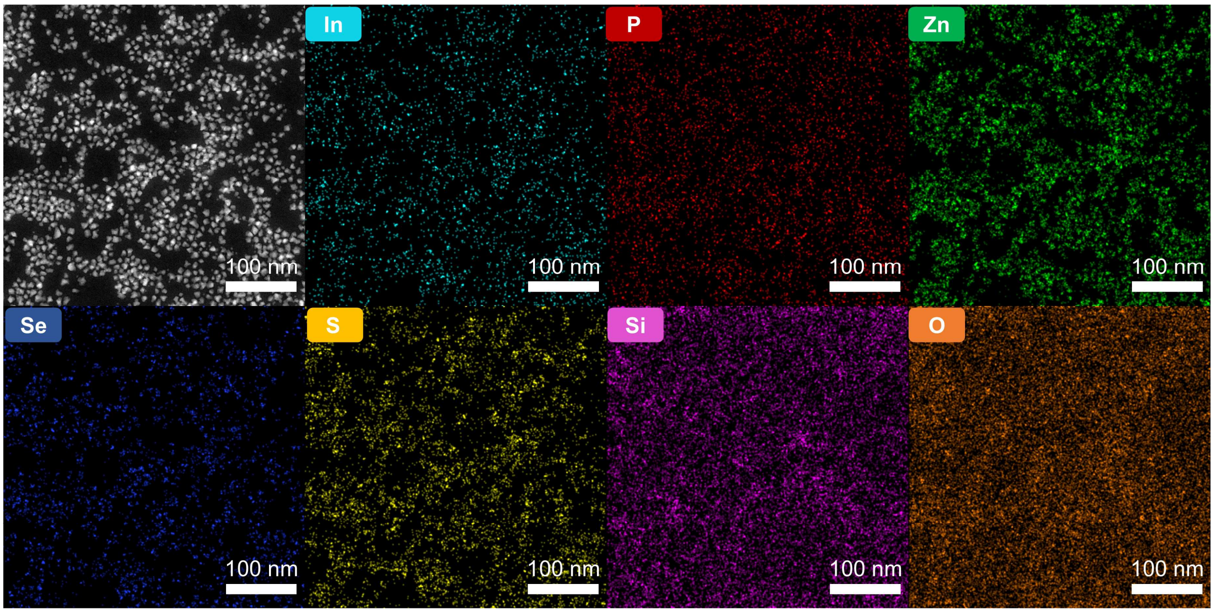

Figure S10. STEM images and EDS mapping of the dried samples from the mixture solution containing UDAC-QDs and PDMS compounds before the curing process.

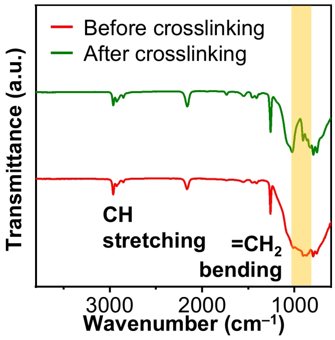

Figure S11. FT-IR spectra of the UDAC-QDs/PDMS B composites before and after curing in the absence of PDMS A.

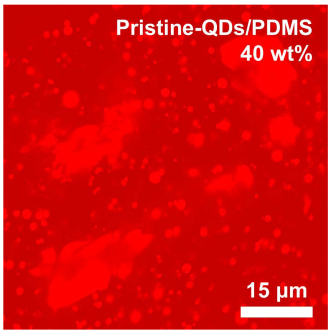

Figure S12. Confocal microscope image of the pristine-QDs/PDMS composite film (QD loading: 40 wt%).

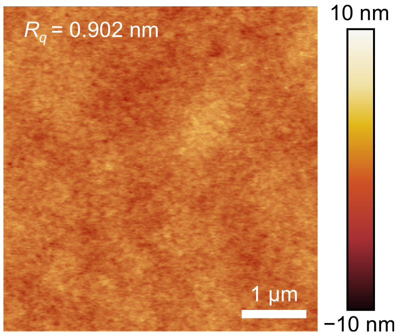

Figure S13. AFM image of UDAC-QDs/PDMS CCLs (root mean square roughness: 0.902 nm).

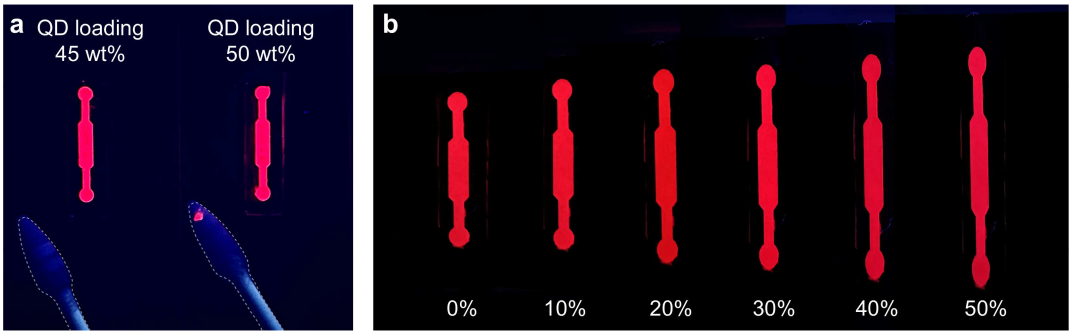

Figure S14. UDAC-QDs/PDMS CCLs with high QD loadings. a) Photographs of UDAC-QDs/PDMS CCLs with different QD loadings (left: 45 wt% QDs, right: 50 wt% QDs). b) Photographs of UDAC-QDs/PDMS CCLs (45 wt% QDs) under uniaxial tensile strain ranging from 0% to 50%.

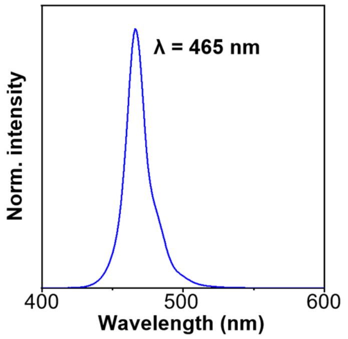

Figure S15. Emission spectrum of blue micro-LEDs employed in this study (λ: 465 nm).

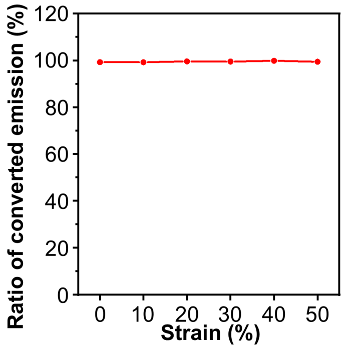

Figure S16. Ratio of converted light emission to total emission of micro-LEDs integrated with UDAC-QDs/PDMS CCLs under varying applied strains.

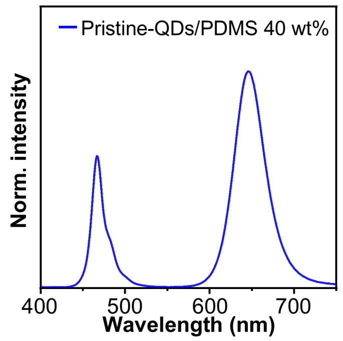

Figure S17. Emission spectrum of micro-LEDs integrated with pristine-QDs/PDMS CCLs (40 wt% QDs).

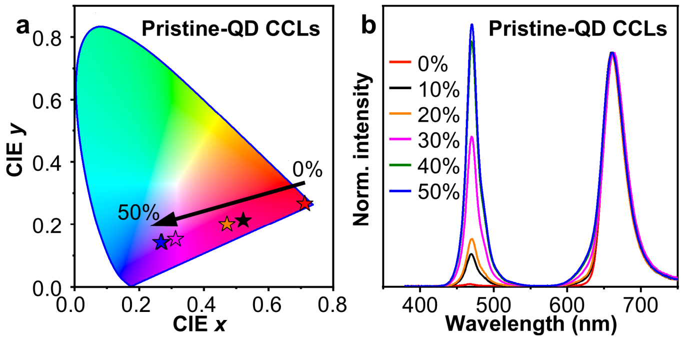

Figure S18. Optical performance of conventional rigid CCLs under applied strains. a,b) (a) CIE color coordinates and (b) emission spectra of micro-LEDs (λ: 465 nm) integrated with rigid QD CCLs based on pristine-QD films under varying applied strains.

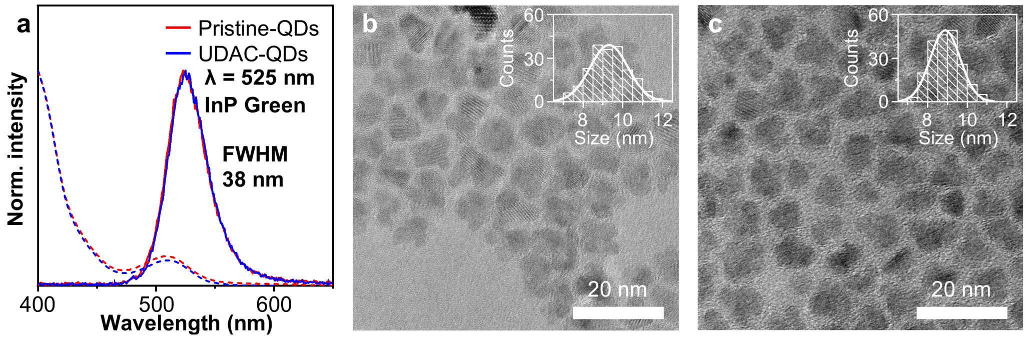

Figure S19. UDAC-functionalization for green-emitting InP/ZnSe/ZnS QDs. a) Absorption and PL spectra of pristine- and UDAC-functionalized green-emitting InP/ZnSe/ZnS QDs. b,c) TEM images of (b) the pristine- and (c) UDAC-QDs. Insets show the size distribution of the QDs (d_{av} = 9.31 ± 0.96 nm and 8.86 ± 0.76 nm for pristine-QDs and UDAC-QDs, respectively; n = 200).

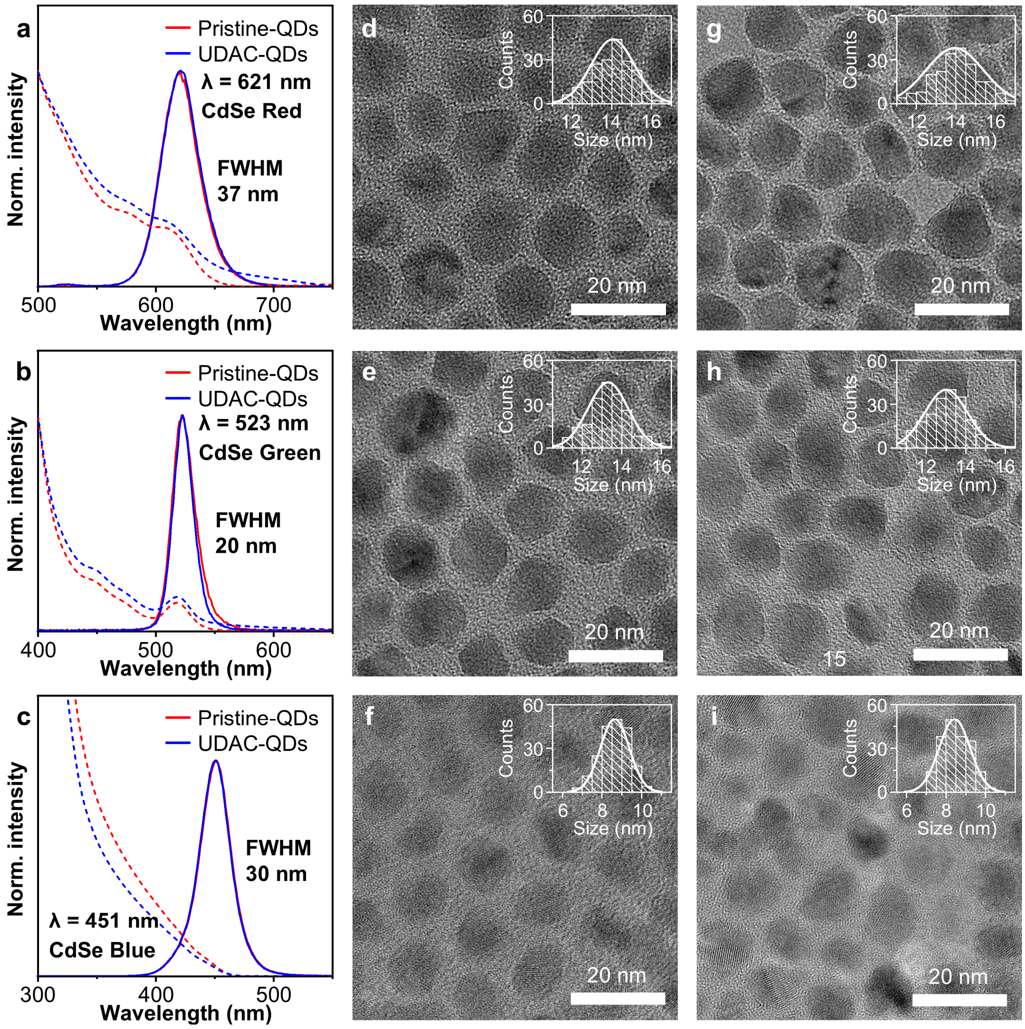

Figure S20. UDAC-functionalization for CdSe-based QDs with various colors. a–c) Absorption and PL spectra of pristine- and UDAC-functionalized (a) red-emitting CdSe@CdZnS QDs, (b) green-emitting CdSe@ZnSe/ZnS QDs, and (c) blue-emitting CdSe@ZnS QDs. d–f) TEM images of the pristine (d) red QDs, (e) green QDs, and (f) blue QDs. g–i) TEM images of the UDAC-functionalized (g) red QDs, (h) green QDs, and blue QDs. Insets show the size distribution of the QDs (d_{av} = 13.92 ± 1.37 nm, 13.94 ± 1.45 nm, 13.20 ± 1.25 nm, 12.90 ± 1.28 nm, 8.63 ± 0.73 nm, and 8.42 ± 0.78 nm for pristine-red QDs, UDAC-red QDs, pristine-green QDs, UDAC-green QDs, pristine-blue QDs, and UDAC-blue QDs, respectively; n = 200).

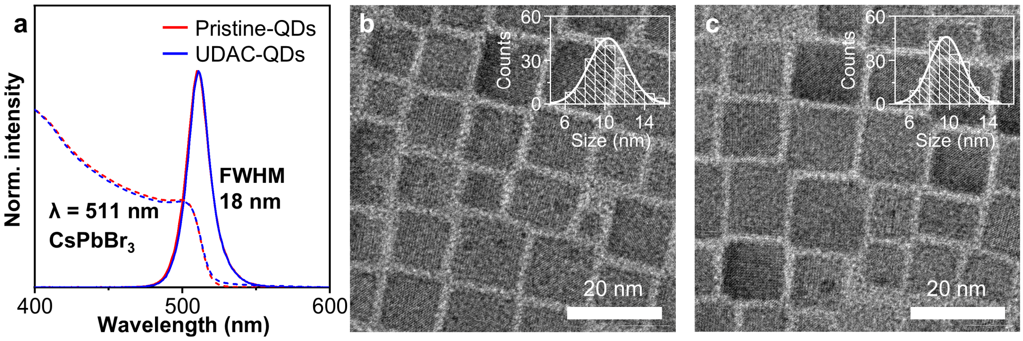

Figure S21. UDAC-functionalization for perovskite QDs. a) Absorption and PL spectra of pristine- and UDAC-functionalized $CsPbBr_3$ QDs. b,c) TEM images of (b) pristine- and (c) UDAC-QDs. Insets show the size distribution of the QDs (d_{av} = 12.36 ± 1.96 nm and 11.68 ± 1.70 nm for pristine-QDs and for UDAC-QDs, respectively; n = 200).

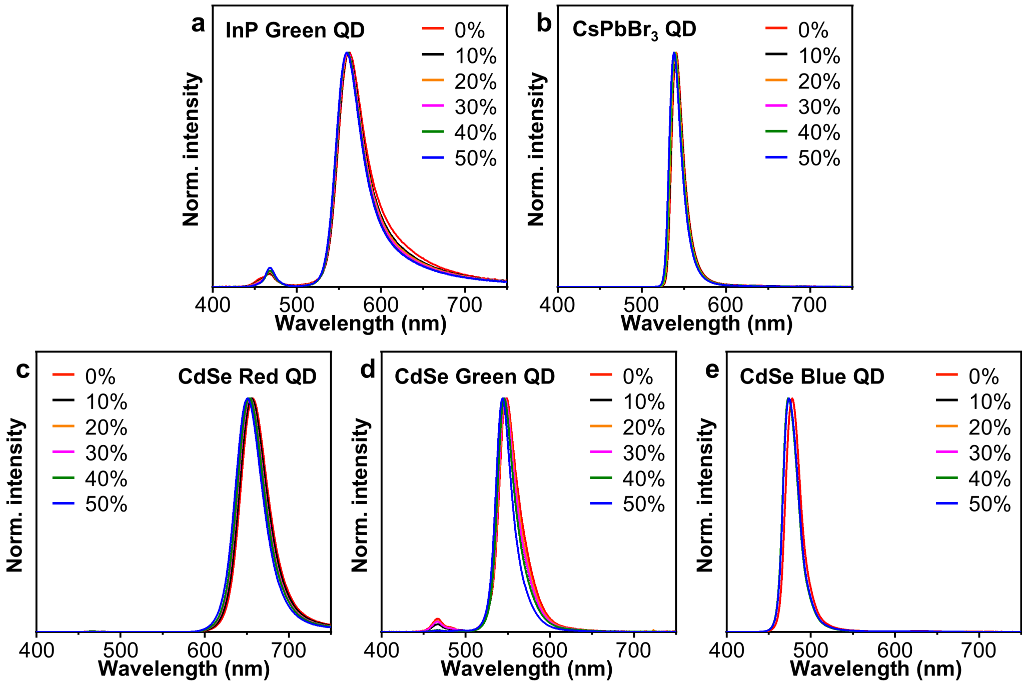

Figure S22. Emission spectra of micro-LEDs employing various UDAC-QDs/PDMS CCLs. a) Green-emitting InP/ZnSe/ZnS QDs, b) green-emitting $CsPbBr_3$ QDs, c) red-emitting CdSe@CdZnS QDs, d) green-emitting CdSe@ZnSe/ZnS QDs, and e) blue-emitting CdSe@ZnS QDs. QD loading was 40 wt% for all CCL samples.

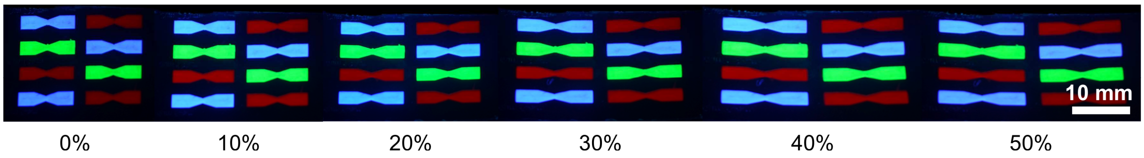

Figure S23. Photographs of the UDAC-QDs/PDMS CCLs under uniaxial tensile strain, ranging from 0% to 50%.

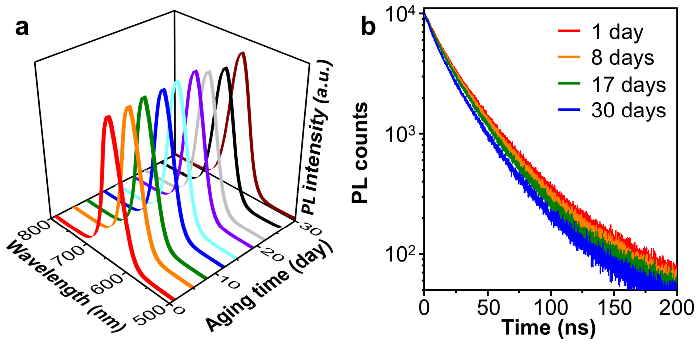

Figure S24. Water stability tests of UDAC-QDs/PDMS CCLs. a,b) (a) PL and (b) time-resolved PL decay spectra of UDAC-QDs/PDMS CCLs after immersion in deionized water for different durations.

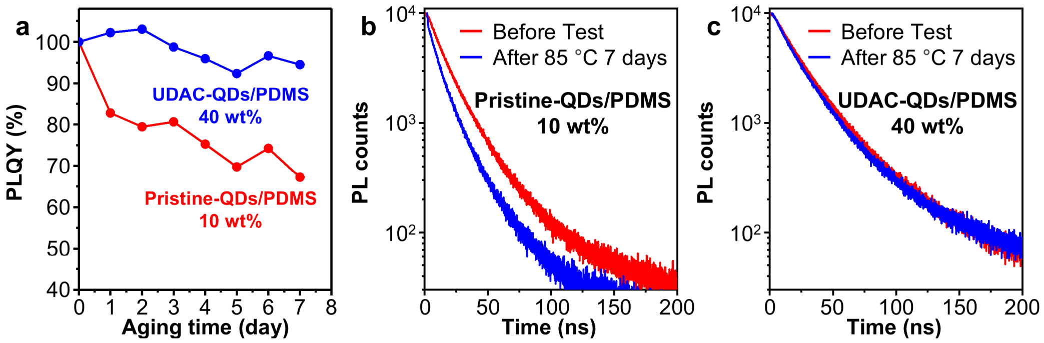

Figure S25. Thermal stability tests of pristine-QDs/PDMS and UDAC-QDs/PDMS CCLs at 85 °C. a) Photoluminescence quantum yield (PLQY) of QDs/PDMS CCLs over time. b,c) Time-resolved PL decay spectra of (b) pristine-QDs/PDMS (10 wt%) and (c) UDAC-QDs/PDMS (40 wt%) CCLs before and after 7 days of thermal treatment.

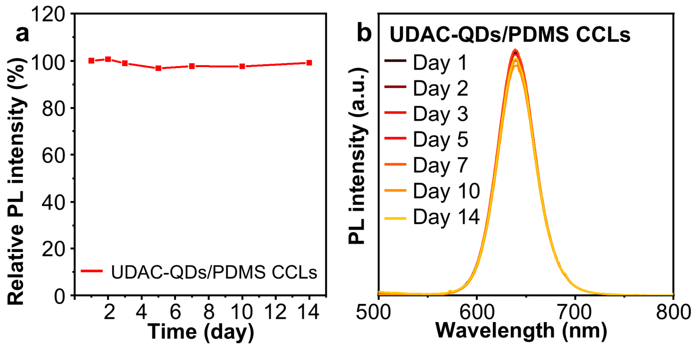

Figure S26. Light stability tests of UDAC-QDs/PDMS CCLs. a) Time-dependent PL intensities and b) PL spectra of UDAC-QDs/PDMS CCLs under continuous UV irradiation (λ: 365 nm).

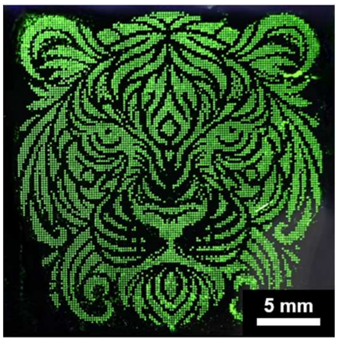

Figure S27. PL image of pixelated QD CCL patterns, displaying a tiger.

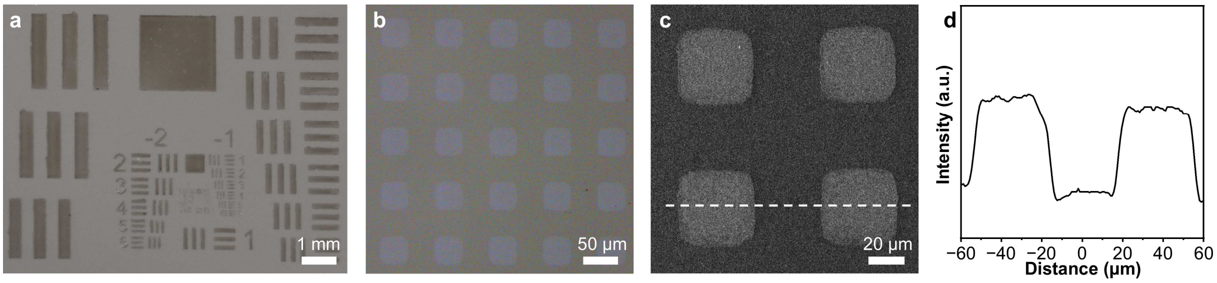

Figure S28. Structural analysis of patterned pixels. a) Optical image of the patterned UDAC-QDs/PDMS pixels of varying sizes. b,c) (b) Optical microscopy image and (c) SEM image of the patterned UDAC-QDs/PDMS pixels (313 PPI). d) SEM line profile along the white dashed line in (c).

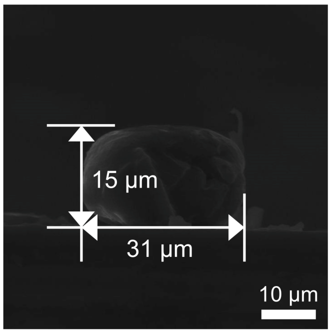

Figure S29. Cross-sectional SEM image of a patterned UDAC-QDs/PDMS pixel.

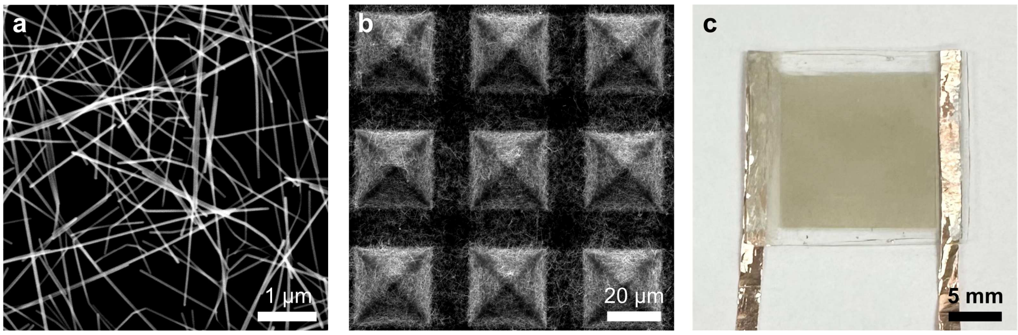

Figure S30. Material and structural characterization of touch sensors. a) SEM image of Ag NWs. b,c) (b) SEM image and (c) photograph of the Ag NW-based pyramid-shaped touch sensor.

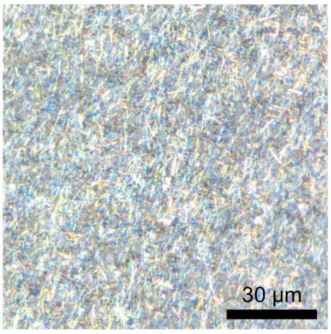

Figure S31. Optical microscopy image of Ag NWs embedded in stretchable PUA matrix.

4. References for the Supporting Information

[S1] M. K. Choi, J. Yang, D. C. Kim, Z. Dai, J. Kim, H. Seung, V. S. Kale, S. J. Sung, C. R. Park, N. Lu, T. Hyeon, D.-H. Kim, *Adv. Mater.* 2018, **30**, 1703279.

[S2] J. I. Kwon, G. Park, G. H. Lee, J. H. Jang, N. J. Sung, S. Y. Kim, J. Yoo, K. Lee, H. Ma, M. Karl, T. J. Shin, M. H. Song, J. Yang, M. K. Choi, *Sci. Adv.* 2022, **8**, eadd0697.

[S3] M. Y.-M. Wong, C. C.-Y. Poon, Y.-T. Zhang, *Cardiovasc. Eng.* 2009, **9**, 32.
